# The complete mitochondrial genome of *Stibochiona nicea* (Gray, 1846) (Lepidoptera: Nymphalidae) and phylogenetic analysis

**DOI:** 10.1080/23802359.2023.2221348

**Published:** 2023-06-09

**Authors:** Hangying Zhang, Qinghe Chen, Qiaoyu Xie, Qinghua Lin, Gang Sun, Yan Fang, Qinghui Shi

**Affiliations:** a School of Resources and Chemical Engineering, Sanming University, Sanming, China; b Fujian Provincial Key Laboratory of Resources and Environment Monitoring & Sustainable Management and Utilization, Sanming University, Sanming, China; c Medical Plant Exploitation and Utilization Engineering Research Center, Sanming University, Sanming, China

**Keywords:** Nymphalidae, *Stibochiona nicea*, mitochondrial genome, phylogeny

## Abstract

In this study, the complete mitochondrial genome (mitogenome) of *Stibochiona nicea* (Gray, 1846) (Lepidoptera: Nymphalidae) was first reported with 15,298 bp in size, containing 13 protein-coding genes (PCGs), 22 tRNA genes, two rRNA genes (*rrnL* and *rrnS*), and one control region. The nucleotide composition of the entire mitogenome is highly A + T biased (81.5%). The gene content and arrangement of the newly sequenced mitogenome are identical to those of the other available mitogenomes of Nymphalidae. All PCGs start with the conventional ATN codons, except for *cox1* initiating with atypical CGA(R). Nine PCGs (*atp8*, *atp6*, *cox3*, *nad1*, *nad2*, *nad3*, *nad4l*, *nad6*, and *cob*) utilize a typical stop codon TAA, whereas the remaining PCGs (*cox1*, *cox2*, *nad4*, and *nad5*) end with an incomplete stop codon T–. Phylogenetic analysis revealed that *S. nicea* is closely related to *Dichorragia nesimachus* within Pseudergolinae, which further forms the sister group to the grouping of (Nymphalinae + (Cyrestinae + (Biblidinae + Apaturinae))). The complete mitogenome of *S. nicea* will provide useful genetic information for improving the taxonomic system and phylogenetics of Nymphalidae.

## 1. Introduction

The family Nymphalidae (Lepidoptera: Papilionoidea) is the species-richest group of butterflies with more than 6100 species distributed on all continents except Antarctica (Ackery et al. 1999; van Nieukerken et al. 2011). The nymphalids have been intensively investigated as model taxa for a wide range of evolutionary and ecological studies due to the distinctive species richness and ecological diversification (Boggs et al. 2003). However, the internal relationships of nymphalids are still controversial, especially regarding the phylogenetic status of Pseudergolinae, Biblidinae, Cyrestinae, and Libytheinae (Wahlberg et al. 2009; Wu et al. 2014; Shi et al. 2015; Espeland et al. 2018; Yang et al. 2020). The insect mitochondrial genomes (mitogenomes) provide effective molecular markers for studies in systematics, population genetics, and evolutionary biology (Cameron 2014; Yang et al. 2020). For Pseudergolinae, only one complete mitogenome (*Dichorragia nesimachus*) is available up to now (Wu et al. 2014), which hinders clarification of the phylogeny of Pseudergolinae. *Stibochiona nicea* (Gray, 1846), commonly known as the popinjay, belongs to Pseudergolinae and is mainly distributed in China, India, Nepal, Sikkim, Bhutan, Vietnam, and Malaysia. The upper side of its wings is black, and the underside is tan. There is a row of neat small white spots on the outer edge of the fore wing, including two white spots on the 2a compartment. In addition, there is a row of white spots on the outer edge of the hind wing, and a row of black spots and blue bands on the inner side of the white spots. To better understand the mitogenomic structure of *S. nicea* and the phylogenetic position of Pseudergolinae, we newly determined the complete mitogenome of *S. nicea* and conducted a phylogenetic analysis of Nymphalidae.

## 2. Materials

**Figure 1.**
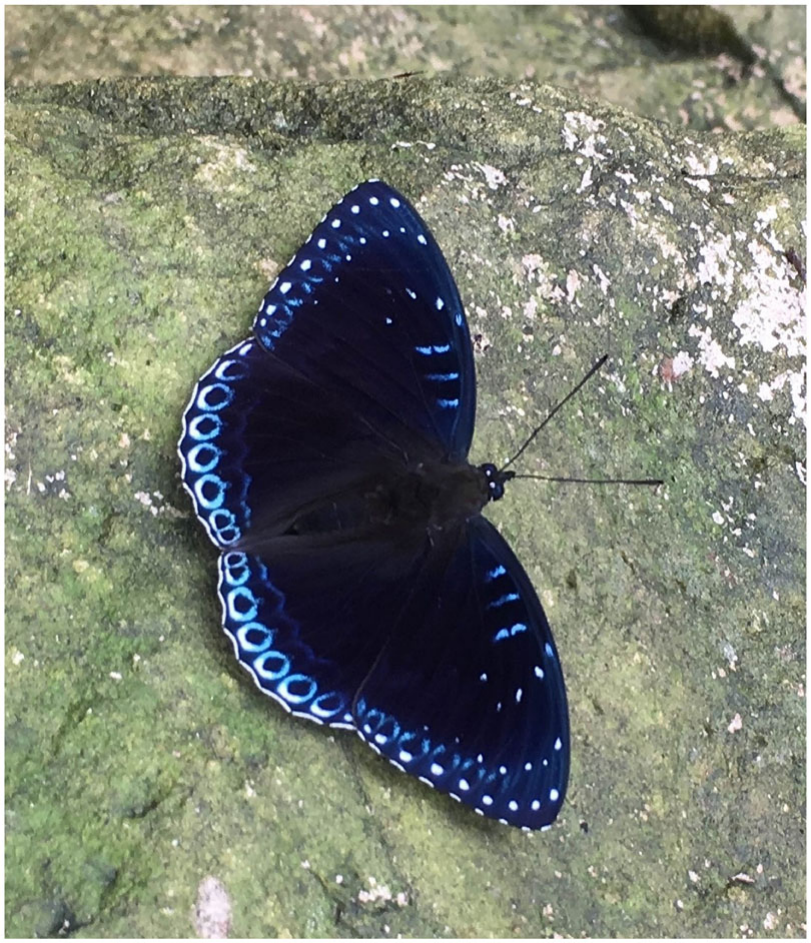
The morphological characteristics of *Stibochiona nicea*. This picture was taken by Qinghui Shi at Minqing County, Fuzhou City, Fujian Province, China.

The specimen of *S. nicea* was collected from Minqing County, Fuzhou City, Fujian Province, China (26.219 N, 118.862 E) in August 2018. A specimen image of *S. nicea* is shown in Figure 1, taken by Qinghui Shi. The fresh individual was preserved in absolute ethyl alcohol and deposited at the Medical Plant Exploitation and Utilization Engineering Research Center, Sanming University (Dr. Qinghui Shi, 214213898@qq.com) under the voucher number SMU-20180824.

## 3. Methods

Total genomic DNA was extracted and purified from thorax muscle of *S. nicea* using the Rapid Animal Genomic DNA Isolation Kit (Sangon Biotech, Shanghai, China) according to the manufacturer’s instructions. The complete mitochondrial sequences, amplified by PCR with 12 pairs of primers (Table S1), were obtained through Sanger sequencing with ABI 3730XL DNA analyzer (Sangon Biotech, Shanghai, China). The PCR products were visualized by electrophoresis on 1.2% agarose gel, and the gel images are shown in Figures S1–S6. The raw sequences were assembled and annotated using the BioEdit 7.0 (Hall 1999) and MEGA 11.0 software (Tamura et al. 2021) with reference to the mitogenome of *D. nesimachus* (GenBank accession no. KF590541). The complete mitogenome of *S. nicea* has been submitted to GenBank database with the accession number MZ458541. The circular-mapping mitogenome of *S. nicea* was plotted using the OGDRAW program (Greiner et al. 2019).

The phylogenetic tree was reconstructed based on concatenated nucleotide sequences of 13 PCGs and two rRNAs of *S. nicea*, other 65 representatives from 12 subfamilies of the Nymphalidae, and two outgroup species from Lycaenidae. The 13 PCGs and two rRNAs were first aligned individually using MEGA 11.0 software (Tamura et al. 2021), then concatenated using DAMBE 7.0 (Xia 2018). The best-fit model (GTR + I + G) was selected for concatenate sequences using jModeltest 2.1.10 (Darriba et al. 2012) under the corrected Akaike information criterion. The maximum-likelihood (ML) analysis was performed using IQ-TREE 1.6.8 (Nguyen et al. 2015). The bootstrap support values of the tree node were evaluated via the bootstrap test with 1000 replicates.

## 4. Results and discussion

**Figure 2.**
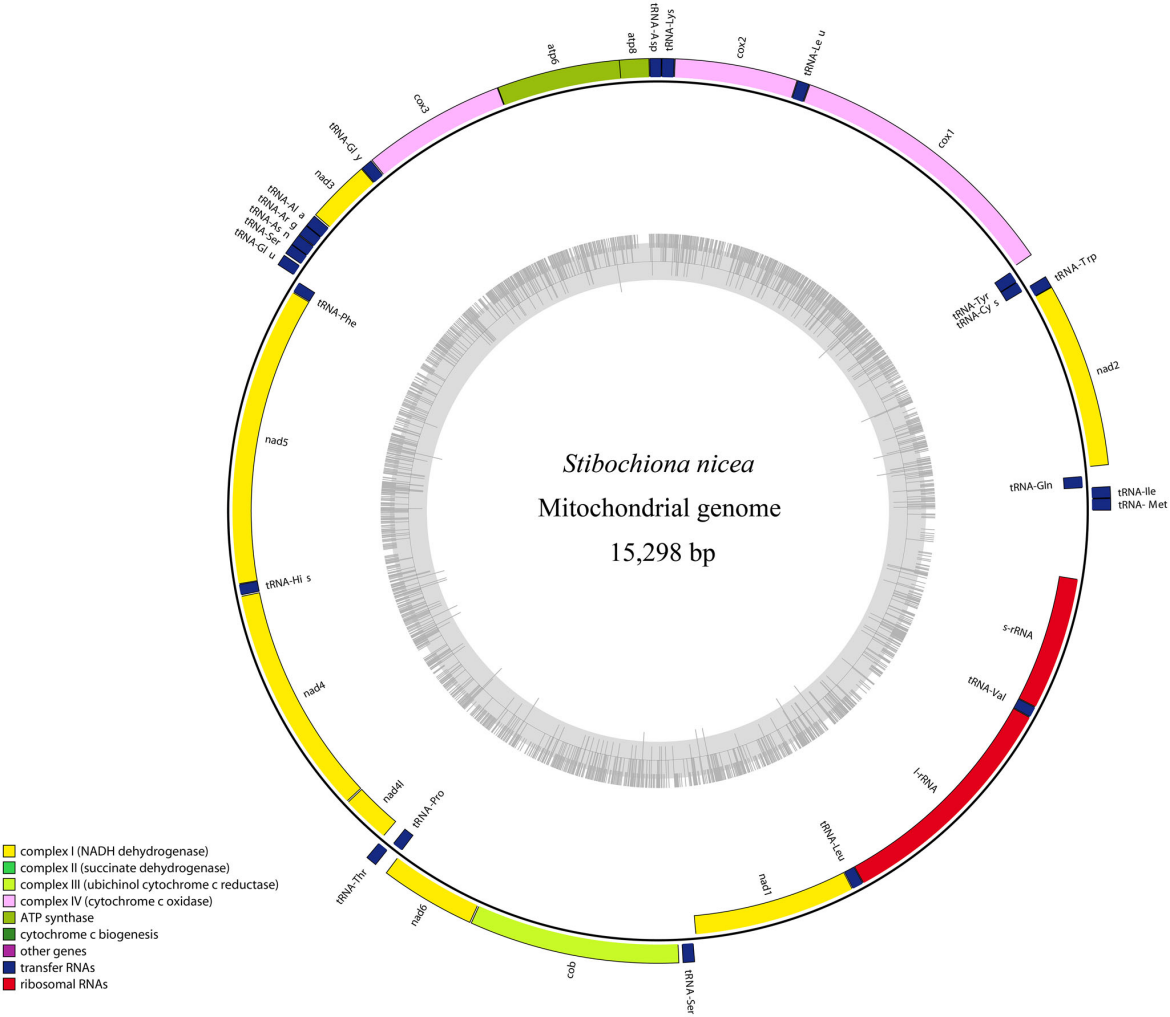
The circular-mapping mitochondrial genome of *Stibochiona nicea*. Gene names on the outside line side indicated that these genes were located on the H-strand, whereas the others were located on the L-strand. Color codes for different genes are listed on the map.

The complete mitogenome of *S. nicea* is a typical circular molecule of 15,298 bp in length, consisting of 13 protein-coding genes (PCGs), 22 tRNA genes, two rRNA genes (*rrnL* and *rrnS*), and one control region (Figure 2). The gene content and arrangement of the *S. nicea* mitogenome are found to be identical to those of the other nymphalid mitogenomes (Wu et al. 2014; Yang et al. 2020). The overall nucleotide contents of A, T, C, and G are 40.0%, 41.5%, 11.0%, and 7.5%, respectively, with a high A + T bias (81.5%).

The total size of the 13 PCGs of *S. nicea* is 11,182 bp accounting for 73.1% of the complete mitogenome, and encoding 3717 amino acids. All PCGs are initiated by typical ATN codons, except for the *cox1* gene, which starts with the unusual CGA as found in most other determined nymphalid mitogenomes (Yang et al. 2020). Four PCGs (*cox1*, *cox2*, *nad4*, and *nad5*) end with an incomplete stop codon T–, while the other PCGs (*atp8*, *atp6*, *cox3*, *nad1*, *nad2*, *nad3*, *nad4l*, *nad6*, and *cob*) terminate with a complete stop codon TAA. All tRNAs can be folded into a typical cloverleaf secondary structure, except for *trnS1*(AGN) lacking the dihydrouridine (DHU) arm, as seen in other reported nymphalids (Wu et al. 2014; Yang et al. 2020). The lengths of *rrnL* and *rrnS* are 1365 bp and 777 bp, respectively. The control region is located between *rrnS* and *trnM* with a length of 392 bp, and contains several structures characteristic of lepidopterans, such as the ATAGA motif followed by a 19 bp poly-T stretch, a microsatellite-like (TA)_9_ element preceded by the ATTTA motif (Salvato et al. 2008; Kim et al. 2014).

**Figure 3.**
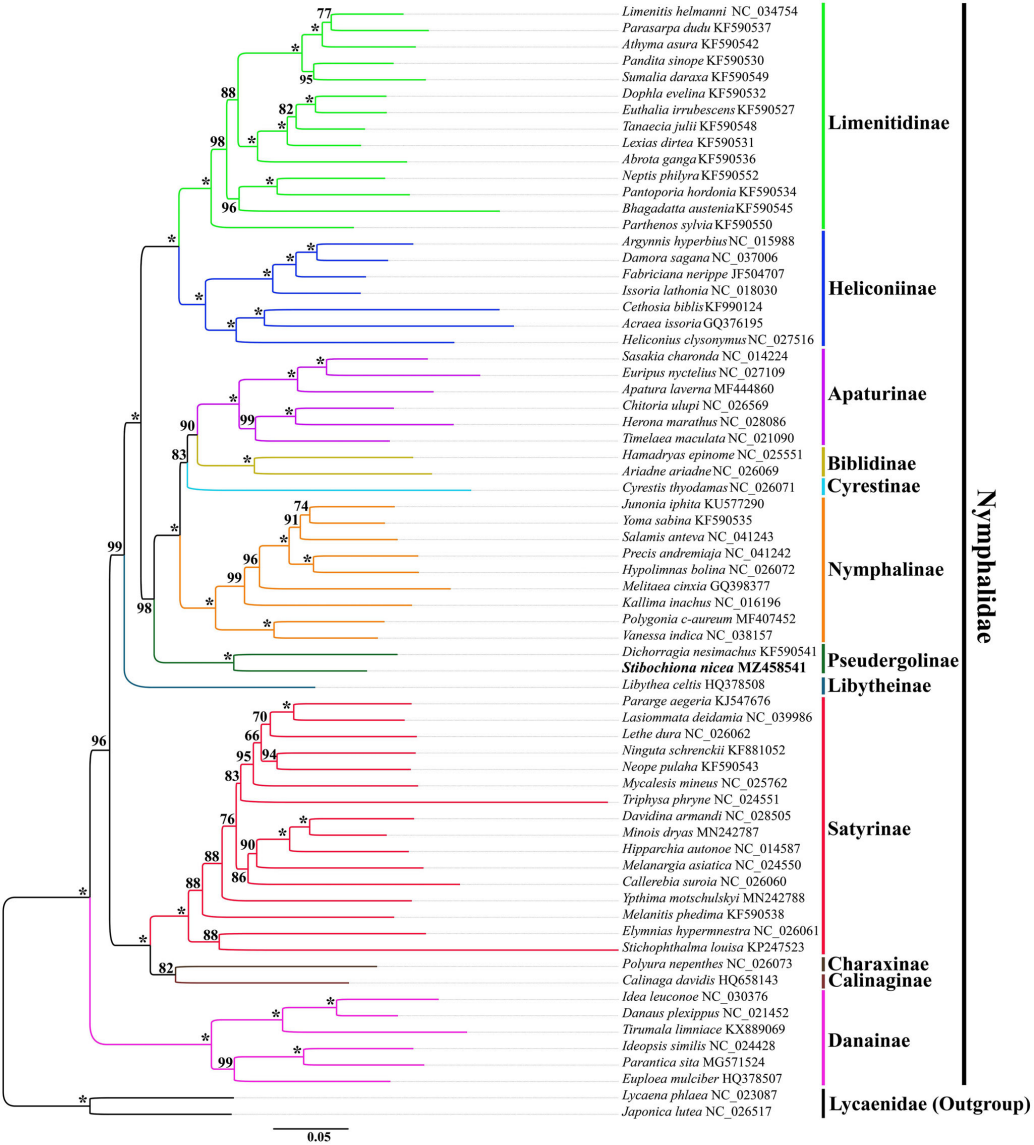
The maximum-likelihood (ML) phylogenetic tree of *Stibochiona nicea* and other nymphalid butterflies. Phylogenetic reconstruction was done from a concatenated matrix of 13 protein-coding mitochondrial genes and two ribosomal RNA genes. The numbers beside the nodes correspond to the bootstrap values based on 1000 replicates (*=100%). Alphanumeric terms indicate the GenBank accession numbers.

In our ML analysis, the phylogenetic relationships among five previously defined Nymphalidae clades are (danaine + (satyrine + (libytheine + (nymphaline + heliconiine)))) (Figure 3), and similar relationships have been established in previous mitogenomic studies (Wu et al. 2014; Shi et al. 2015; Yang et al. 2020). Meanwhile, the phylogenetic tree suggested that *S. nicea* is clustered with *D. nesimachus* within Pseudergolinae, which is sister to other subfamilies of the nymphaline clade with strong bootstrap support. In addition, the phylogenetic relationships among remaining subfamilies of the nymphaline clade are as follows: (Nymphalinae + (Cyrestinae + (Biblidinae + Apaturinae))), which is consistent with that reported recently (Yang et al. 2020). However, different taxon and molecular markers have identified various phylogenetic relationships, including (Nymphalinae + ((Cyrestinae + Biblidinae) + Apaturinae)) (Shi et al. 2015); ((Apaturinae + Biblidinae) + (Cyrestinae + Nymphalinae)) (Wahlberg et al. 2009; Espeland et al. 2018). Therefore, the phylogenetic relationships among these subfamilies of Nymphalidae are still undefined and need to be explored by more comprehensive genome sampling in future studies.

## 5. Conclusions

In the present study, the complete mitogenome of *S. nicea* was assembled and analyzed. We found that the gene content and arrangement of the newly sequenced mitogenome are similar to those of other determined mitogenomes of Nymphalidae. The complete mitogenome of *S. nicea* will provide important information for improving the taxonomic system and phylogenetics of Nymphalidae.

## Supplementary Material

Supplemental Material

Supplemental Material

Supplemental Material

Supplemental Material

Supplemental Material

Supplemental Material

Supplemental Material

Supplemental Material

Supplemental Material

Supplemental Material

Supplemental Material

Supplemental Material

Supplemental Material

## Data Availability

The genome sequence data that support the findings of this study are openly available in GenBank of NCBI at https://www.ncbi.nlm.nih.gov/nuccore/MZ458541 under the accession no. MZ458541.
