# Supplementary material for: The complete mitochondrial genome of Stibochiona nicea (Gray, 1846) (Lepidoptera: Nymphalidae) and phylogenetic analysis
Source: Mitochondrial DNA B Resour. 2023 Jun 9;8(6):648–52. doi: 10.1080/23802359.2023.2221348 (PMC10259338; doi:10.1080/23802359.2023.2221348)
Supplement: Supplemental Material [file TMDN_A_2221348_SM9773.docx]

Table S1. List of primers used for the amplification of *Stibochiona nicea* mitogenome.

| **Gene/Region** | **Primer pair** | **Primer sequence (5’→3’)** | **AT* (℃)** |
| --- | --- | --- | --- |
| *cox1* | SN1F | GGTCAACAAATCATAAAGATATTG | 50.0 |
|  | SN1R | TAAACTTCAGGGTGACCAAAAAAT |  |
| *cox1→cox3* | SN2F | GCTCAGTATTACCTCCATC | 45.6 |
|  | SN2R | CAAGAGTATTCCTCATCG |  |
| *cox3* | SN3F | GTTCTGAGATTTCAGGTAA | 49.6 |
|  | SN3R | TTACTAATAAATCATTTGC |  |
| *cox3→nad5* | SN4F | GAAGGATTACGATGAGGA | 45.4 |
|  | SN4R | AAGTCGGATTGGTGATG |  |
| *nad5* | SN5F | AAAACTTCCAGAAAATAATCTC | 46.5 |
|  | SN5R | TTGCTTTATCTACTTTAAGACA |  |
| *nad5→cob* | SN6F | CTCCGATTTGATAGTGC | 46.2 |
|  | SN6R | ATAAGTGTCTGGGTCTCCT |  |
| *cob* | SN7F | TATGTACTACCATGAGGACAAATAT | 47.0 |
|  | SN7R | ATTACACCTCCTAATTTATTAGGAAT |  |
| *cob→rrnL* | SN8F | CCTTTTAGGAGACCCAGAT | 45.8 |
|  | SN8R | AGACCTTAGGGATAACAGC |  |
| *rrnL* | SN9F | CTGTACAAAGGTAGCATA | 49.0 |
|  | SN9R | GCCAAAACTTTAGTCTAG |  |
| *rrnL→rrnS* | SN10F | GCAGTGTTACGCTGTTATC | 47.8 |
|  | SN10R | CGGTCGTTTAGAGGAATC |  |
| *rrnS* | SN11F | CTTCTACTTTGTTACGACTTA T | 50.0 |
|  | SN11R | AATTTTGTGCCAGCAGTTG |  |
| *rrnS→cox1* | SN12F | CCTACAATACCGCCATA | 48.2 |
|  | SN12R | TCATCCTGTTCCTGCTC |  |

*: Annealing temperature
