# Supplementary figures and images for: The complete mitochondrial genome of Stibochiona nicea (Gray, 1846) (Lepidoptera: Nymphalidae) and phylogenetic analysis
Source: Mitochondrial DNA B Resour. 2023 Jun 9;8(6):648–52. doi: 10.1080/23802359.2023.2221348 (PMC10259338; doi:10.1080/23802359.2023.2221348)

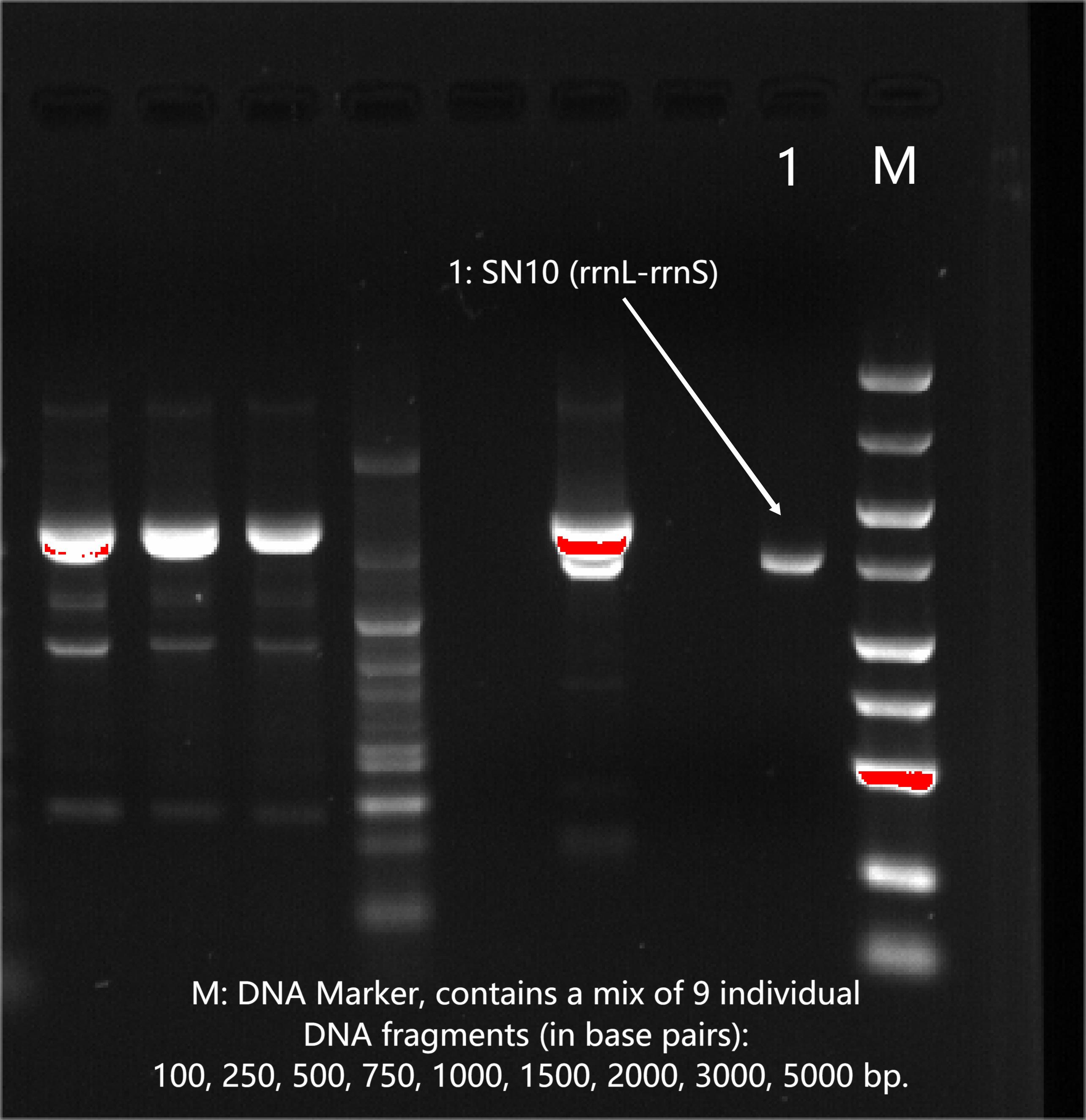

Supplement: Supplemental Material [file TMDN_A_2221348_SM9772.jpg]

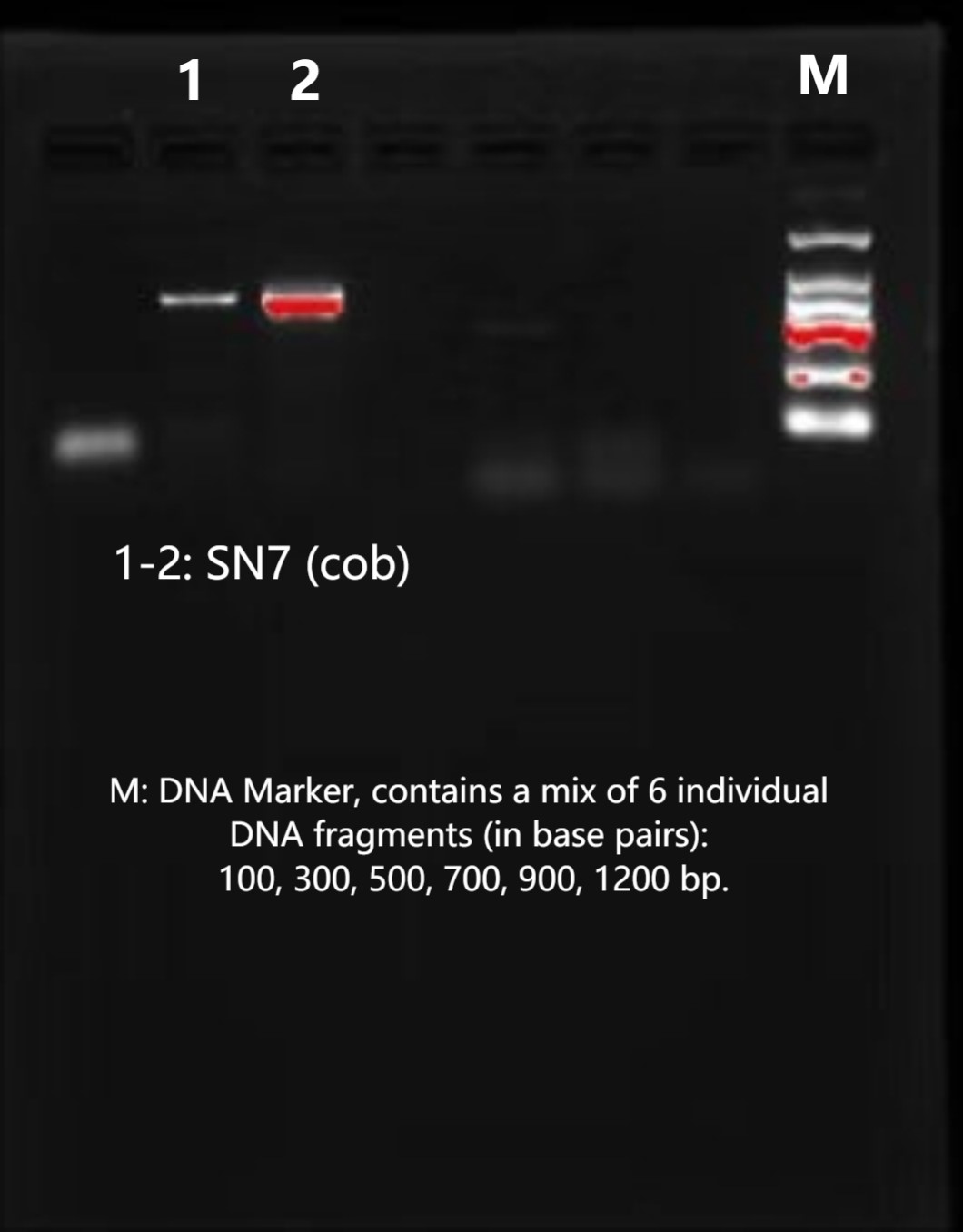

Supplement: Supplemental Material [file TMDN_A_2221348_SM9768.jpg]

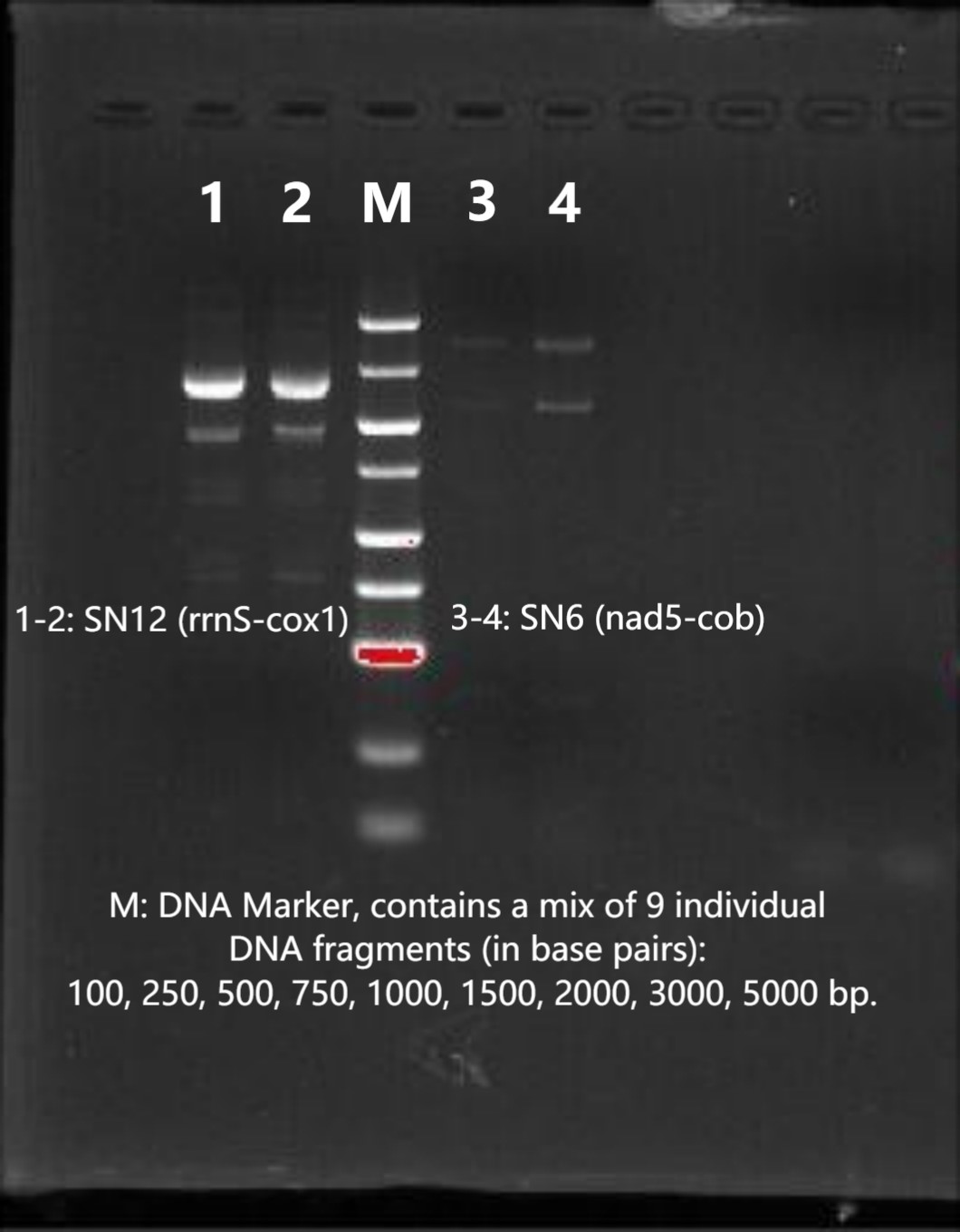

Supplement: Supplemental Material [file TMDN_A_2221348_SM9762.jpg]

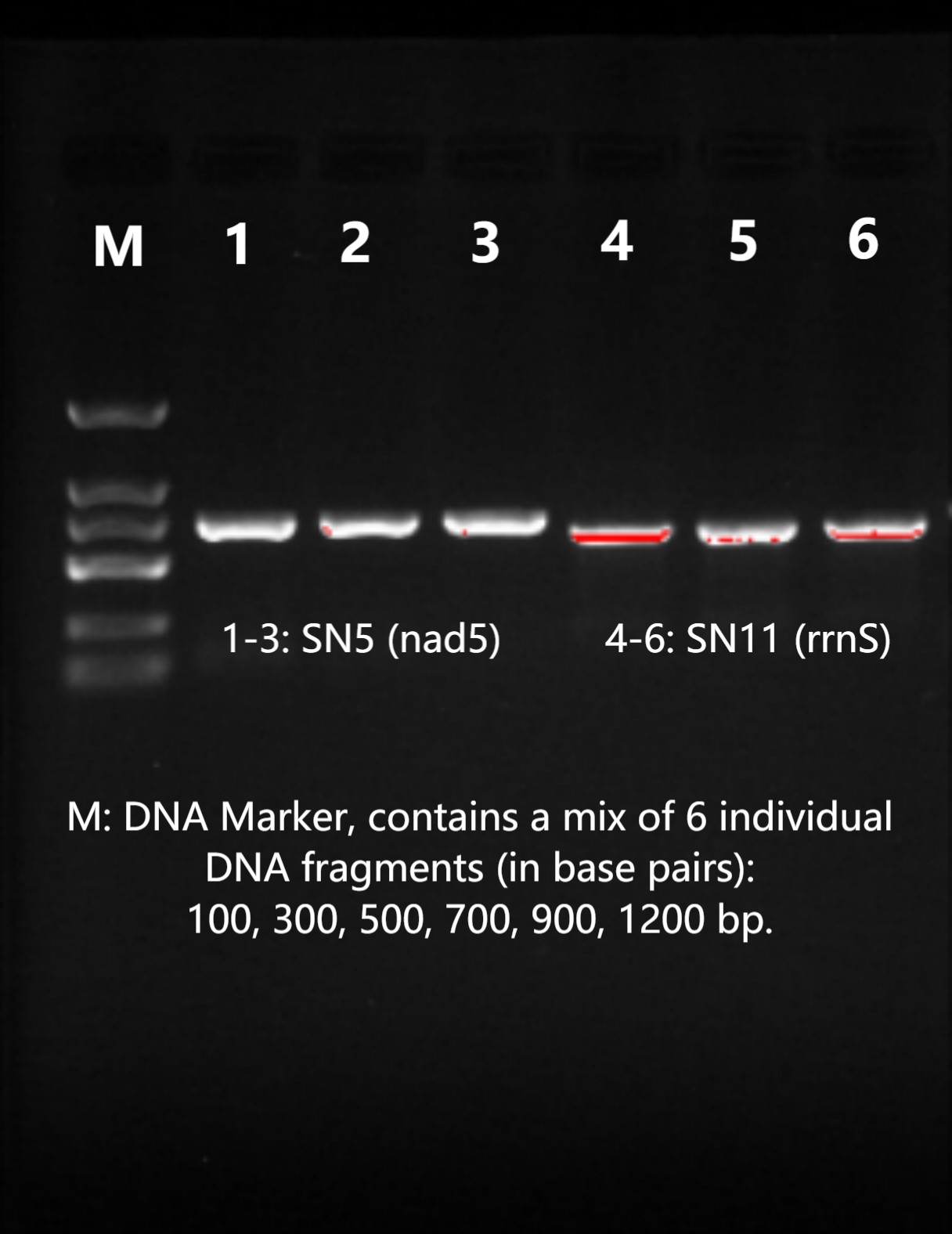

Supplement: Supplemental Material [file TMDN_A_2221348_SM9755.jpg]

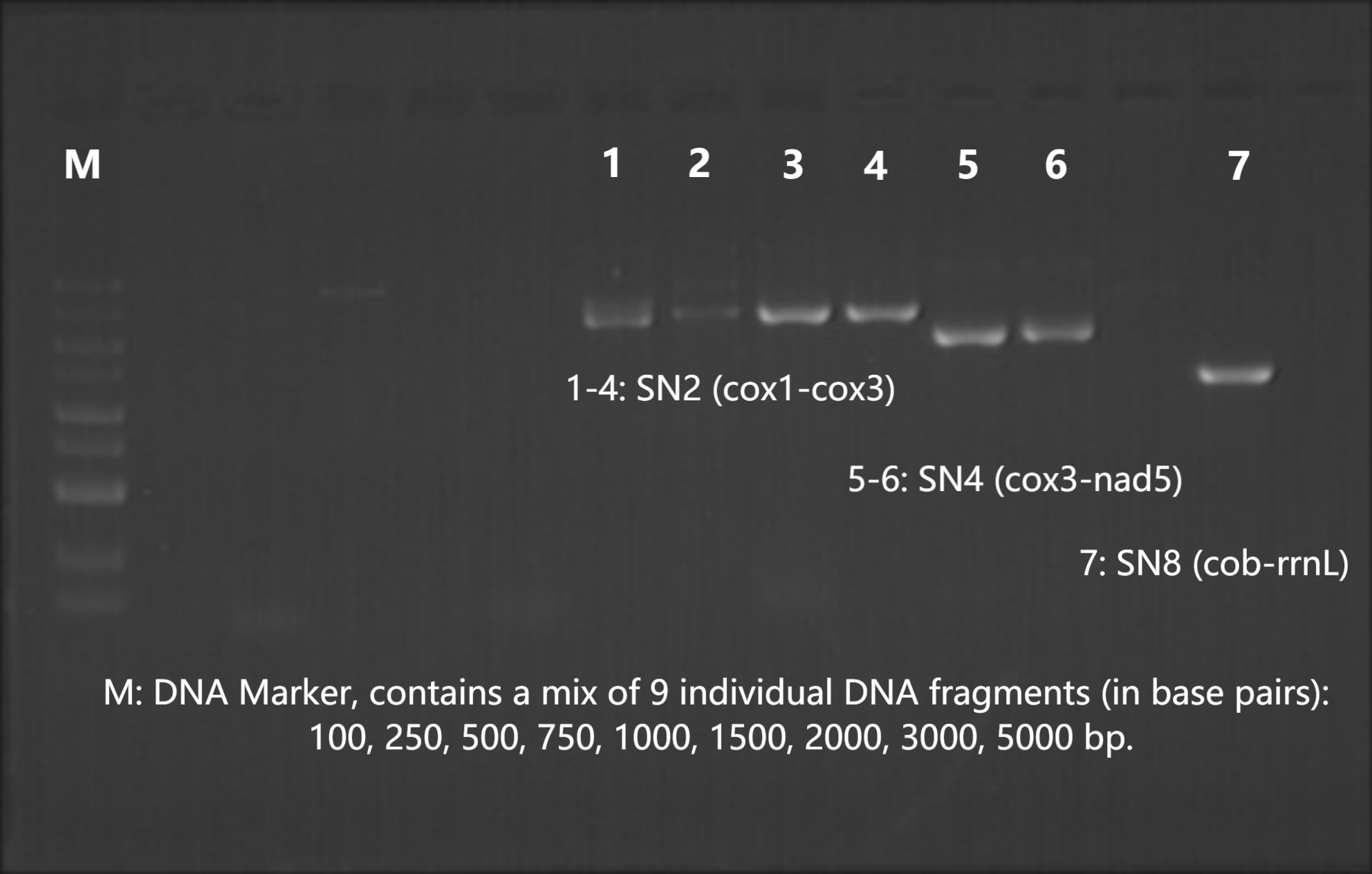

Supplement: Supplemental Material [file TMDN_A_2221348_SM9749.jpg]

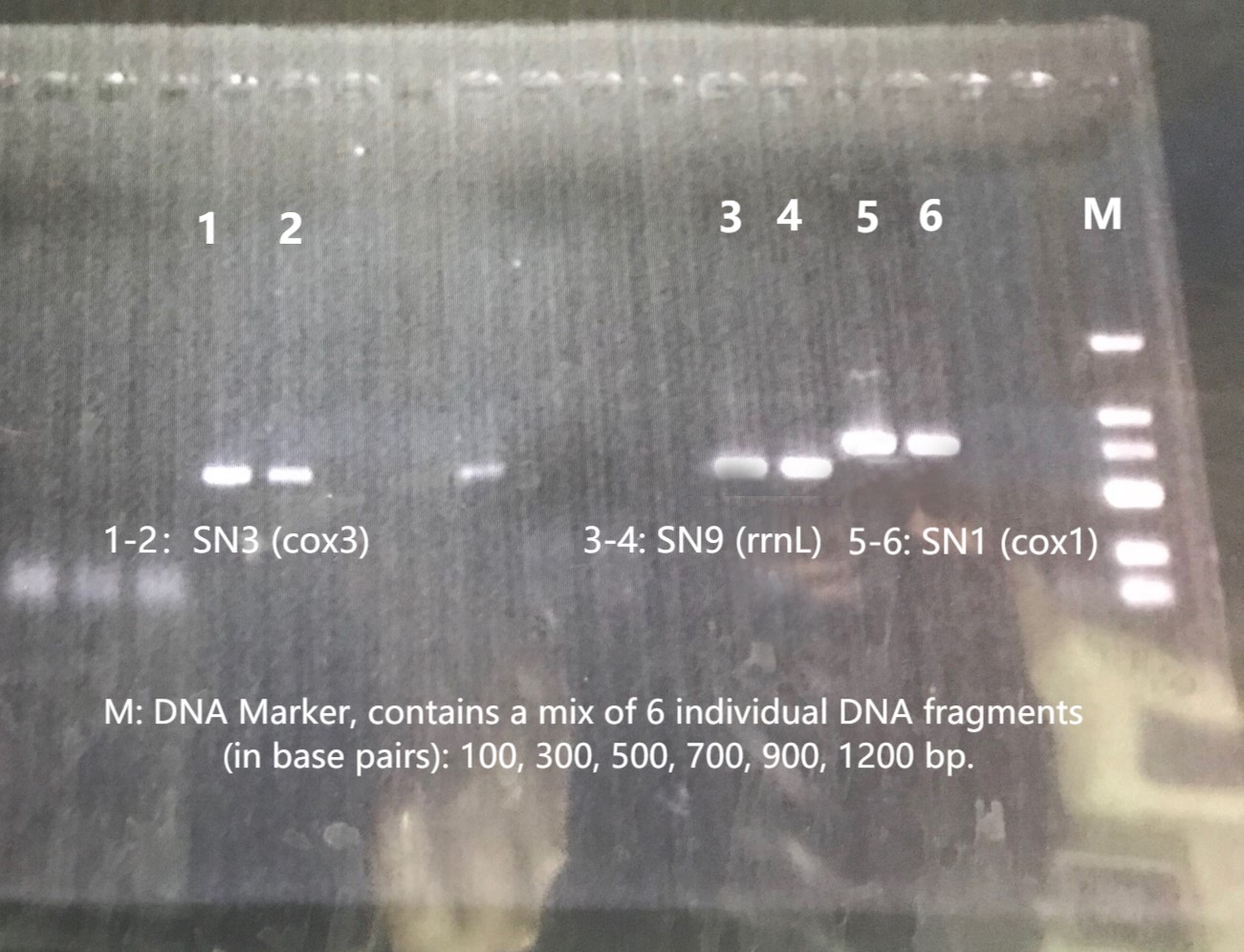

Supplement: Supplemental Material [file TMDN_A_2221348_SM9739.jpg]
